# Pronuclear transfer rescues poor embryo development of *in vitro*-grown secondary mouse follicles

**DOI:** 10.1093/hropen/hoae009

**Published:** 2024-02-10

**Authors:** Antonia Christodoulaki, Haitang He, Min Zhou, Chloë De Roo, Machteld Baetens, Tine De Pretre, Muhammad Fakhar-I-Adil, Björn Menten, Ann Van Soom, Dominic Stoop, Annekatrien Boel, Björn Heindryckx

**Affiliations:** Ghent-Fertility and Stem cell Team (G-FaST), Department for Reproductive Medicine, Ghent University Hospital, Ghent, Belgium; Department of Human Structure and Repair, Ghent University Hospital, Ghent, Belgium; Ghent-Fertility and Stem cell Team (G-FaST), Department for Reproductive Medicine, Ghent University Hospital, Ghent, Belgium; Department of Human Structure and Repair, Ghent University Hospital, Ghent, Belgium; Department of Obstetrics and Gynaecology, Union Hospital, Tongji Medical College, Huazhong University of Science and Technology, Wuhan, China; Ghent-Fertility and Stem cell Team (G-FaST), Department for Reproductive Medicine, Ghent University Hospital, Ghent, Belgium; Department of Human Structure and Repair, Ghent University Hospital, Ghent, Belgium; Ghent-Fertility and Stem cell Team (G-FaST), Department for Reproductive Medicine, Ghent University Hospital, Ghent, Belgium; Department of Human Structure and Repair, Ghent University Hospital, Ghent, Belgium; Department for Reproductive Medicine, Ghent University Hospital, Ghent, Belgium; Department of Biomolecular Medicine, Center for Medical Genetics Ghent (CMGG), Ghent University Hospital, Ghent, Belgium; Department of Biomolecular Medicine, Center for Medical Genetics Ghent (CMGG), Ghent University Hospital, Ghent, Belgium; Ghent-Fertility and Stem cell Team (G-FaST), Department for Reproductive Medicine, Ghent University Hospital, Ghent, Belgium; Department of Human Structure and Repair, Ghent University Hospital, Ghent, Belgium; Department of Biomolecular Medicine, Center for Medical Genetics Ghent (CMGG), Ghent University Hospital, Ghent, Belgium; Faculty of Veterinary Medicine, Department of Reproduction, Obstetrics and Herd Health, University of Ghent, Merelbeke, Belgium; Ghent-Fertility and Stem cell Team (G-FaST), Department for Reproductive Medicine, Ghent University Hospital, Ghent, Belgium; Department of Human Structure and Repair, Ghent University Hospital, Ghent, Belgium; Department for Reproductive Medicine, Ghent University Hospital, Ghent, Belgium; Ghent-Fertility and Stem cell Team (G-FaST), Department for Reproductive Medicine, Ghent University Hospital, Ghent, Belgium; Department of Human Structure and Repair, Ghent University Hospital, Ghent, Belgium; Ghent-Fertility and Stem cell Team (G-FaST), Department for Reproductive Medicine, Ghent University Hospital, Ghent, Belgium; Department of Human Structure and Repair, Ghent University Hospital, Ghent, Belgium

**Keywords:** IVG, follicle development, nuclear transfer, oocyte quality, fertility preservation, embryo development

## Abstract

**STUDY QUESTION:**

Is pronuclear transfer (PNT) capable of restoring embryo developmental arrest caused by cytoplasmic inferiority of *in vitro*-grown (IVG) mouse oocytes?

**SUMMARY ANSWER:**

PNT to *in vivo* matured cytoplasm significantly improved embryo development of IVG mouse oocytes, leading to living, fertile offspring.

**WHAT IS KNOWN ALREADY:**

*In vitro* follicle culture has been considered as a fertility preservation option for cancer patients. Studies describing the culture of human follicles remain scarce, owing to low availability of tissue. Mouse models have extensively been used to study and optimize follicle culture. Although important achievements have been accomplished, including the production of healthy offspring in mice, IVG oocytes are of inferior quality when compared to *in vivo*-grown oocytes, likely because of cytoplasmic incompetence.

**STUDY DESIGN, SIZE, DURATION:**

The study was carried out from September 2020 to February 2022. In total, 120 15-day-old B6D2 mice were used to perform secondary follicle culture and assess the quality of IVG oocytes. *In vivo*-grown control oocytes were obtained from 85 8- to 12-week-old B6D2 mice, following ovarian stimulation. For sperm collection, four B6D2 males between 10 and 14 weeks old were used. For embryo transfer, 14 8- to 12-week-old CD1 females served as surrogate mothers and 10 CD1 vasectomized males 10–24 weeks old were used to generate pseudo-pregnant females. Finally, for mating, four B6D2 female mice aged 8–10 weeks and two B6D2 male mice aged 10 weeks old were used to confirm the fertility of nuclear transfer (NT)-derived pups.

**PARTICIPANTS/MATERIALS, SETTING, METHODS:**

Secondary follicles from 15-day-old B6D2 mice were isolated from the ovaries and cultured for 9 days, before a maturation stimulus was given. Following 16–18 h of maturation, oocytes were collected and evaluated on maturation rate, oocyte diameter, activation rate, spindle morphology, calcium-releasing ability, and mitochondrial membrane potential. For every experiment, *in vivo*-grown oocytes were used as a control for comparison. When cytoplasmic immaturity and poor embryo development were confirmed in IVG oocytes, PNT was performed. For this, the pronuclei from IVG oocytes, created following parthenogenetic activation and IVF, were transferred to the cytoplasm of fertilized, *in vivo*-grown oocytes. Genetic analysis and embryo transfer of the generated embryos were implemented to confirm the safety of the technique.

**MAIN RESULTS AND THE ROLE OF CHANCE:**

Following 9 days of follicle culture, 703 oocytes were collected, of which 76% showed maturation to the metaphase II stage. Oocyte diameters were significantly lower in IVG oocytes, measuring 67.4 μm versus 73.1 μm in controls (*P* < 0.001). Spindle morphology did not differ significantly between IVG and control oocytes, but calcium-releasing ability was compromised in the IVG group. An average calcium release of 1.62 arbitrary units was observed in IVG oocytes, significantly lower than 5.74 in control oocytes (*P* < 0.001). Finally, mitochondrial membrane potential was inferior in IVG compared to the control group, reaching an average value of 0.95 versus 2.27 (*P* < 0.001). Developmental potential of IVG oocytes was assessed following parthenogenetic activation with strontium chloride (SrCl_2_). Only 59.4% of IVG oocytes cleaved to two cells and 36.3% reached the blastocyst stage, significantly lower than 89.5% and 88.2% in control oocytes, respectively (*P* < 0.001 and 0.001). Both PNT and spindle transfer (ST) were explored in pilot experiments with parthenogenetically activated oocytes, as a means to overcome poor embryo development. After the added value of NT was confirmed, we continued with the generation of biparental embryos by PNT. For this purpose, IVG and control oocytes first underwent IVF. Only 15.5% of IVG oocytes were normally fertilized, in contrast to 45.5% in controls (*P* < 0.001), with resulting failure of blastocyst formation in the IVG group (0 versus 86.2%, *P* < 0.001). When the pronuclei of IVG zygotes were transferred to the cytoplasm of control zygotes, the blastocyst rate was restored to 86.9%, a similar level as the control. Genetic analysis of PNT embryos revealed a normal chromosomal profile, to a rate of 80%. Finally, the generation of living, fertile offspring from PNT was possible following embryo transfer to surrogate mothers.

**LARGE-SCALE DATA:**

N/A.

**LIMITATIONS, REASONS FOR CAUTION:**

Genetic profiles of analysed embryos from PNT originate from groups that are too small to draw concrete conclusions, whilst ST, which would be the preferred NT approach, could not be used for the generation of biparental embryos owing to technical limitations. Even though promising, the use of PNT should be considered as experimental. Furthermore, results were acquired in a mouse model, so validation of the technique in human IVG oocytes needs to be performed to evaluate the clinical relevance of the technology. The genetic profiles from IVG oocytes, which would be the ultimate characterization for chromosomal abnormalities, were not analysed owing to limitations in the reliable analysis of single cells.

**WIDER IMPLICATIONS OF THE FINDINGS:**

PNT has the ability to overcome the poor cytoplasmic quality of IVG mouse oocytes. Considering the low maturation efficiency of human IVG oocytes and potential detrimental effects following long-term *in vitro* culture, NT could be applied to rescue embryo development and could lead to an increased availability of good quality embryos for transfer.

**STUDY FUNDING/COMPETING INTEREST(S):**

A.C. is a holder of FWO (Fonds voor Wetenschappelijk Onderzoek) grants (1S80220N and 1S80222N). B.H. and A.V.S. have been awarded with a special BOF (Bijzonder Onderzoeksfonds), GOA (Geconcerteerde onderzoeksacties) 2018000504 (GOA030-18 BOF) funding. B.H. has been receiving unrestricted educational funding from Ferring Pharmaceuticals (Aalst, Belgium). The authors declare that they have no conflict of interest.

WHAT DOES THIS MEAN FOR PATIENTS? The growth of follicles, which contain the oocytes (eggs), by *in vitro* culture has been proposed as a fertility preservation option for patients with cancer, but it remains an experimental approach. Owing to the limited access to human follicles and ovarian tissue, several animal models have been studied to optimize follicle culture and assess the quality of *in vitro*-grown (IVG) oocytes. Promising results have been reported by several groups around the world using the mouse, and these studies have even resulted in living offspring. Nevertheless, the quality of IVG oocytes is inferior to that of oocytes that develop in the body (*in vivo*). In this study, we applied ‘nuclear transfer’ (NT) to improve embryo development of IVG oocytes. NT is a technique that involves transfer of the genetic material of an oocyte or zygote to the cytoplasm of a counterpart, which has had the genetic material removed. The genetic material (pronuclei) from IVG mouse zygotes were transferred to the cytoplasm of zygotes from *in vivo*-grown oocytes, and this led to increased embryo rates and even to live births. These results are promising for the application and safety of NT, and indicate that this technology could be used to overcome poor oocyte-related quality. Ultimately, this technique could be considered for IVG oocytes from cancer patients, to increase the number of available embryos for transfer and to overcome the detrimental effects of long-term *in vitro* follicle culture on oocyte quality.

## Introduction

Women are born with a pre-determined number of follicles, which decreases over the years, until the end of reproductive life, with approximately just 1000 follicles remaining in the ovaries at menopause (Kim *et al.*, 2016). Any disruption in the initial follicular population may lead to premature ovarian insufficiency and infertility (Goswami and Conway, 2005). During cancer treatment, the use of chemotherapy or radiotherapy (acting through different mechanisms) may lead to premature activation or apoptosis of the different follicular stages, causing follicular exhaustion (Meirow *et al.*, 2010; Jones *et al.*, 2017). Since these therapies might impair fertility, guidance is necessary for cancer patients (Lambertini *et al.*, 2016).

Current approaches for female fertility preservation include ovarian stimulation for vitrification of oocytes/embryos or cryopreservation of ovarian tissue followed by transplantation after remission of the disease (Andersen *et al.*, 2019; Anderson *et al.*, 2020; Dolmans *et al.*, 2021). Vitrification of oocytes collected following oophorectomy has also been reported in recent years, leading to promising results and live births (Hourvitz *et al.*, 2015; Segers *et al.*, 2015, 2020; Kirillova et al., 2021). However, these options cannot be offered to all patients. In cases where cancer treatment needs to be initiated quickly, ovarian stimulation might not be an option (Practice Committee of the American Society for Reproductive Medicine, 2019), while for certain patients, there is a risk of re-introducing malignant cells following ovarian tissue transplantation (Donnez *et al.*, 2010).

Alternatively, the growth of follicles *in vitro* has been proposed as a female fertility preservation strategy (Herta *et al.*, 2018). This approach is of special interest since a high number of follicles can be isolated during the process of ovarian tissue cryopreservation (Kristensen *et al.*, 2011) and potentially lead to the generation of mature oocytes available for vitrification. Nonetheless, *in vitro* follicle growth still remains experimental. In humans, the results of follicular growth remain poor compared to animal models, owing to the scarcity of the material. So far, only a few groups have achieved the generation of mature oocytes from human follicles grown *in vitro*, and with limited production of mature oocytes (Xiao *et al.*, 2015; McLaughlin *et al.*, 2018; Xu *et al.*, 2021), but functional assessment of these oocytes is still lacking.

Several animal models have been described where follicle culture was successful (Herta *et al.*, 2018), including non-human primates (Xu *et al.*, 2011), but the most promising results originate from mice. Mouse offspring have been generated from *in vitro*-grown (IVG) follicles since 1996, initiating from the most immature stage, primordial follicles (Eppig *et al.*, 1996). Over the years, this protocol has been adapted (O’Brien *et al.*, 2003) and several culture systems have been developed (Simon *et al.*, 2020), allowing the growth of mouse follicles completely *in vitro* from several stages of folliculogenesis (Xu *et al.*, 2006; Jin *et al.*, 2010; Laronda *et al.*, 2017; Matsushige *et al.*, 2022; Taghizabet *et al.*, 2022).

Despite these great advances, IVG oocytes show inferior oocyte activation and embryo development rates compared to their *in vivo*-grown counterparts (Kim *et al.*, 2004; Takashima *et al.*, 2021; Xu and Zelinski, 2022). In addition, recent studies have shown that the metabolic, transcriptomic and epigenetic profiles of IVG oocytes differ from that of *in vivo*-grown oocytes (Kim *et al.*, 2004; Saenz-de-Juano *et al.*, 2020; Herta *et al.*, 2022).

For this study, we cultured secondary mouse follicles. Our objective was not to improve current follicle culture systems, but to investigate whether nuclear transfer (NT) could be applied for the indication of poor embryo development in order to maximize embryo growth and assess the safety of the technology. NT is a technique that involves the transfer of the genetic material of an oocyte or zygote to the cytoplasm of an enucleated counterpart (Craven *et al.*, 2017). Several NT techniques have been developed, but the most studied ones are spindle transfer (ST) and pronuclear transfer (PNT) (Christodoulaki *et al.*, 2021). To date, one healthy baby has been born after the application of ST to overcome mitochondrial DNA diseases (Zhang *et al.*, 2017), while lately it has also been considered as a means to overcome certain infertility indications, such as fertilization failure and embryo developmental arrest in mice and human (Costa-Borges *et al.*, 2020; Tang *et al.*, 2020, 2022; Costa-Borges *et al.*, 2023). Finally, the use of NT for female-related infertility recently led to the first live births in human, at a very high efficiency (Costa-Borges* et al.*, 2023).

In this study, we assessed the cytoplasmic quality of IVG mouse oocytes and aimed to increase their developmental potential by performing PNT. With this approach, we were able to improve the development of the IVG embryos to levels equal to controls, leading to living, fertile offspring. Our observations provide important evidence that PNT could be applied as means to overcome poor oocyte cytoplasmic quality.

## Materials and methods

### Ethical approval and animal housing

This study was approved by the Animal Ethics Committee of Ghent University Hospital (ECD no. 19/60 and ECD 19/60aanv). Animals were housed in the central animalarium of the Ghent University Hospital, were fed *ad libitum* and were exposed to 12-h light–dark cycles. B6D2 females and B6D2 males were purchased from Janvier Laboratories (Le Genest-Saint-Isle, France) and CD1 females and vasectomized CD1 males from Charles River laboratories (Saint-Germain-Nuelles, France).

### Isolation of follicles and *in vitro* culture

Secondary follicles measuring ∼110–130 μm in diameter were isolated from 15-days-old female B6D2 mice, using 26 gauge needles (VWR, Leuven, Belgium). Follicles were first collected in Leibovitz’s L-15 medium (Fisher Scientific, Aalst, Belgium), supplemented with 100 IU/100 μg/ml penicillin/streptomycin (Life Technologies Europe NV, Ghent, Belgium) and 10% heat-inactivated foetal bovine serum (FBS, Fisher Scientific). Next, follicles were washed in two central wells of pre-incubated growth medium (GM) and placed in Corning V-bottom 96-well plates (Merck Life Science, Hoeilaart, Belgium), in a volume of 75 μl. GM consisted of a-MEM medium + GlutaMAX (Fisher Scientific), supplemented with 5 μg/ml insulin, 5 μg/ml transferrin, 4 ng/ml selenium (Merck Life Science), 5% heat-inactivated FBS, 100 IU/100 μg/ml pen/strep and 10 mIU/ml recombinant FSH (Merck Life Science). Half of the medium was replaced every second day. Follicles were cultured for 9 days in the incubator, under 37°C, 6% CO_2_, and 5% O_2_.

### Oocyte maturation

On Day 9 of follicle culture, GM was replaced with IVM medium. Maturation medium consisted of GM supplemented with 4 ng/ml epidermal growth factor (EGF, Roche, Vilvoorde, Belgium) and 1.2 IU/ml hCG (Pregnyl, Organon, Brussels). Following 16–18 h of maturation, cumulus oocyte complexes (COCs) were used for IVF or denuded with hyaluronidase (200 IU/ml, Merck Life Science) for 1 min and evaluated for their maturation status. For the control oocytes (*in vivo*-grown group), we recruited metaphase II (MII) oocytes after stimulation of B6D2 female mice (8–12 weeks old). An injection of 7.5 IU pregnant mare’s serum gonadotrophin (Folligon, MSD AH, Brussels, Belgium) was given, followed by a second injection with 7.5 IU hCG (Chorulon, MSD AH) 48 h apart. Following 12–14 h of the Chorulon injection, mice were euthanized by cervical dislocation and COCs were collected from the ovarian ampulla. Complexes were either used for IVF or denuded with hyaluronidase and used at the MII stage.

### Spindle–chromosome-complex staining

For spindle–chromosome-complex staining, all chemicals were purchased from Sigma (Merck Life Science), unless stated otherwise. Briefly, MII oocytes were fixed in microtubule-stabilizing buffer (0.1 M PIPES, 5 mM MgCl_2_, 2.5 mM EGTA, 0.01% aprotinin, 1 mM dithiothreitol, 50% deuterium oxide, 1 pM taxol, 0.1% Triton X-100 and 3% formalin) for 30 min. Following this, oocytes were exposed to mouse primary antibodies for a- (1/200) (T9026) and b-tubulin (1/200) (T5293) overnight, at 4°C. Next, they were subjected to secondary antibody (Alexa fluor 594 Donkey anti-mouse, ab150108 Abcam, Cambridge, UK) for 2 h at room temperature. Staining of chromosomes was performed with Hoechst-33258 for 1 h at room temperature. After every step, oocytes were washed extensively three times. For imaging, oocytes were placed in droplets of HEPES in glass bottom dishes (WillCo Wells BV, Amsterdam, The Netherlands). Images were taken with a confocal microscope (Zeiss LSM9000, Zaventem, Belgium). Details of confocal microscopy were as follows: objective lens: 40× with oil, numerical aperture: 1.3, magnification: 400×, type of detector: Multialkali-photomultiplier tube (PMT), pinhole size: red channel: 1 arbitrary unit (AU)/37 μm, blue channel: 1 AU/28 μm, bright field: 1.60 AU, and emission bandpass detection: red channel: 594: 580–700 V, blue channel: 400–580 V, bright field: 400–400 V. Imaging conditions were as follows: laser power: red channel: 2.10%, blue channel: 1.40%, bright field: 2.10%, and pixel-dwell time: 16.08 μs, detector gain and offset: red channel: gain: 650 V, offset: 0, blue channel: 937 V, offset: 0, bright field: 464 V, offset: 0 V.

### Calcium imaging

Calcium imaging was performed as previously described (Bonte *et al.*, 2020). Briefly, oocytes were exposed to KSOM medium supplemented with 7.5 mM Fura 2-AM (Teflabs, Austin, TX, USA) for 30 min. Fura is a Ca^2^^+^-sensitive dye that binds to intracellular Ca^2^^+^. Oocytes were activated with SrCl_2_ and transferred to an inverted epifluorescence microscope (Olympus IX71, Olympus, Antwerp, Belgium), under standard culture conditions (37°C, 6% CO_2_, 5% O_2_). Calcium release was recorded for two consecutive hours with a 10× objective and a filter switch (Lambda DG-4 filter switch, Sutter Instrument Company, Novato, CA, USA) to provide excitation alternating between 340 and 380 nm. Calcium data were analysed using Clampfit 10.2 software (Molecular Devices LLC, San Jose, CA, USA). The total amount of calcium released (in AU) was calculated as the product of the mean amplitude (maximum fluorescence intensity 340/380 nm per peak) per mean frequency (number of calcium spikes) for all oocytes analysed per condition (including the oocytes showing no calcium peaks).

### Mitochondrial membrane potential staining

In order to assess mitochondrial membrane potential, oocytes were exposed to Invitrogen JC-1 staining (Fisher Scientific). JC-1 is a potential dependant, dual emission dye that accumulates in mitochondria and emits green or red fluorescence. When mitochondria membrane potential (ΔΨm) is >140 mV, JC-1 forms aggregates and emits red fluorescence (emission: 590 nm). On the contrary, when ΔΨm is <100 mV, JC-1 remains a monomer and emits green fluorescence (emission: 529 nm). Oocytes were incubated in 15 μg/ml JC-1 staining in small groups for 10 min, at 37°C. Oocytes were then washed and imaged with a confocal microscope (Zeiss LSM9000). Z-stack images were taken for every oocyte. To evaluate the mitochondrial membrane potential, Z-stack images were merged and the ratio of red/green fluorescence was calculated using ImageJ (National Institutes of Health, Bethesda, MD, USA). Detailed microscopy conditions were the following: objective lens: 20×, with 2.8 scanning zoom, numerical aperture: 0.5, magnification: 560×, type of detector: Multialkali-PMT, pinhole size: red channel: 1 AU/47 μm, green channel: 1 AU/38 μm, emission bandpass detection: red channel: 534–700 V, green channel: 400–538 V. Imaging conditions: laser power: red channel: 0.3%, green channel: 0.6%, pixel-dwell time: 8.82 μs, detector gain and offset: red channel: gain: 702 V, offset: 0, green channel: gain: 736 V, offset: 0. Z-stack details: number of slices: 10, stack interval: 5 μm.

### Parthenogenetic activation

For parthenogenetic activation, IVG and control MII oocytes were exposed to Ca^2^^+^-free KSOM medium, supplemented with 10 mM SrCl_2_ and 2 μg/ml cytochalasin D for 4 h.

### Spindle transfer

MII oocytes were first exposed for 10 min in HEPES medium containing 1 μg/ml cytochalasin D, on a heated (37°C) microscope plate, before manipulation. First, oocytes serving as cytoplasmic recipients were enucleated after a hole was made in the zona pellucida with a laser objective. The spindle was visualized with an OosightMeta System (Hamilton Thorne, Beverly, MA, USA) in an Olympus IX71 inverted microscope and removed using a 15-μm enucleation pipette (Cooper Surgical, Venlo, The Netherlands). The same technique was performed for the spindle donors, and the spindle was transferred into the cytoplasmic recipient, after exposing it for 10 s to Hemagglutinin virus of Japan envelop (HVJ-E) (Bioconnect, Huissen, The Netherlands). The spindle was left in the perivitelline space of the recipient oocytes and fusion occurred within an average of 10 min. Fusion was confirmed using the OosightMeta system.

### IVF

For IVF, frozen sperm from 9- to 13-week-old B6D2 male mice was utilized. Sperm straws were warmed at 37°C in a water bath for 10 min and released in a 90-μl drop of FertiUp medium (Bioconnect, Huissen, The Netherlands). The sample was left in the incubator for at least 30 min before use. COCs from IVG and control groups were placed in CARD medium (Bioconnect) droplets and exposed to 12 μl of warmed sperm. Complexes were incubated with sperm for 4 h and then washed in mHTF medium (Bioconnect).

### Pronuclear transfer

PNT was performed following parthenogenetic activation or IVF. Following parthenogenetic activation, pronuclei (PNs) could be seen after 5 h. Parthenogenetic zygotes were exposed for 10 min at 37°C in HEPES medium supplemented with 1 μg/ml Cytochalasin D and 1 μg/ml Nocodazole. In the same way, as described for ST, PNs from the DNA donor zygotes were transferred using a biopsy pipette of 22 μm (Cooper Surgical, Venlo, The Netherlands) to zygote cytoplasm previously enucleated from the PNs. Fusion was performed with HVJ-E. Reconstructed zygotes were placed in mHTF medium overnight and transferred to KSOM medium the next morning.

For the generation of biparental embryos following IVF, PNs were visible following 5–6 h after sperm incubation. Only normally fertilized zygotes (presence of two PNs and second polar body (PB)) were used for PNT.

### Embryo development

Embryos from control, IVG, parthenogenetic embryos and reconstructed zygotes were cultured in KSOM medium until Day 2.5, when the medium was exchanged with Sydney IVF Blastocyst Medium (Cook Medical, Limerick, Ireland). Embryos were cultured up to Day 3.5, under standard culture conditions (37°C, 6% CO_2_, 5% O_2_).

### Embryo vitrification/warming

IVF and reconstructed PNT embryos were vitrified on Day 3.5 using the Vit Kit—Freeze NX kit (Irvine Scientific, Tilburg, The Netherlands), according to the manufacturer’s instructions. Warming was performed using the Vit Kit—Warm NX kit (Irvine Scientific). Following warming, embryos were let to recover for 2 h before embryo transfer or genetic evaluation were performed.

### Embryo transfer

Embryo transfer was performed with a non-surgical catheter system (NSET Device, Bio Services BV, Uden, The Netherlands). CD1 female mice between 8 and 12 weeks were placed in bedding with CD1 male mice for 3 days. The oestrus phase was confirmed by vaginal morphology. Females demonstrating oestrus induction were mated overnight with vasectomized CD1 male mice aged 10–24 weeks. Mating was confirmed next morning by the presence of a vaginal plug. Warmed embryos were transferred with the help of a catheter into the womb of female mice, 3 days post-coitum. Following approximately 18–19 days of gestation, live pups were born.

### Mating of animals

Following embryo transfer, PNT-pups were kept until adulthood and were mated with male and female B6D2 mice. Male and female mice were kept together in the same cage (one female with one male) until pregnancy was confirmed. The male mice were then removed from the cage to avoid further mating.

### Genetic analysis

In order to detect copy number variations (CNVs), we performed shallow whole genome sequencing, as previously described (Tang *et al.*, 2020). Whole blastocysts were snap-frozen in 2 μl of 1× PBS before undergoing whole genome amplification with a SurePlex DNA Amplification System (Rubicon Genomics Inc., Ann Arbor, MI, USA). Next-generation sequencing was performed as described previously (Tang *et al.*, 2020). Briefly, a NEXTflex™ Rapid DNA-Seq Library Prep Kit for Illumina Sequencing (Bioo Scientific, Uden, The Netherlands) was used for library preparation and Agencourt AMPure XP beads for purification (Beckman Coulter, Suarlée, Belgium). Preparation of template was performed on the cBot™ System (Illumina, San Diego, CA, USA) using 2.5 nM of equimolar pooled libraries. Sequencing was performed on Hiseq3000 (Illumina). Data analysis on CNVs was performed using the WisecondorX and Vivar software (CMGG, Ghent, Belgium).

### Statistical analysis

Comparison between categorical variables expressed in percentages (%) was performed with the chi-square statistical test (χ^2^). Average AU values expressing total calcium release and intensity from mitochondrial staining were analysed using Mann–Whitney’s non-parametric test. Oocyte diameters were compared using Student’s *t*-test for independent samples. Statistical significance was set at *P* < 0.05. Statistical analysis was performed with the SPSS Statistics programme (version 27, IBM Corp., New York, USA).

## Results

### Nuclear maturation is possible in IVG-cultured follicles

**Figure 1.**
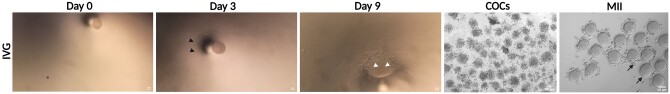
**Complete *in vitro* mouse follicle culture from Day 0 up to the release of mature oocytes**. Follicle development of IVG oocytes from B6D2 15-day-old mice: Day 0: secondary follicle measuring 110–130 μm in diameter. Scale bar: 50 μm. Day 3: follicle on Day 3, still retaining the basal membrane. Proliferation of theca cells is evident (black arrowheads). Scale bar: 50 μm. Day 9: Diffused follicle before the maturation stimulus. A corona of cumulus cells is formed around the oocyte (white arrowheads). Scale bar: 50 μm. COCs: Cumulus oocyte complexes from IVG oocytes collected following the maturation stimulus. Scale bar: 50 μm. MII: mature IVG oocytes, with the first polar body (black arrows). Scale bar: 50 μm. IVG: *in vitro*-grown, COCs: cumulus oocyte complexes, MII: metaphase II.

In total, 1547 secondary follicles measuring between 110 and 130 μm in diameter were isolated on Day 0 (Fig. 1). Following 3 days of culture (Day 3), follicles showed growth and theca cells attached on the dish, with evident proliferation. By Day 9, the basal membrane ruptured and the granulosa cells of the follicle were diffused, but no clear antral formation was observed. Nevertheless, formation of corona cells was evident in 913/1547 (59%) of the follicles. Follicles with a distinct corona around the oocyte were stimulated with EGF and hCG. A total of 703/913 (77%) COCs were released from the follicles 16–18 h later (Day 10). The COCs were treated with hyaluronidase and were evaluated for their maturation status. In total, 534/703 (76%) of the oocytes reached the MII stage, as confirmed by the release of the first polar body (Fig. 1).

### Oocyte growth is suboptimal in IVG oocytes, but spindle formation is not affected

**Figure 2.**
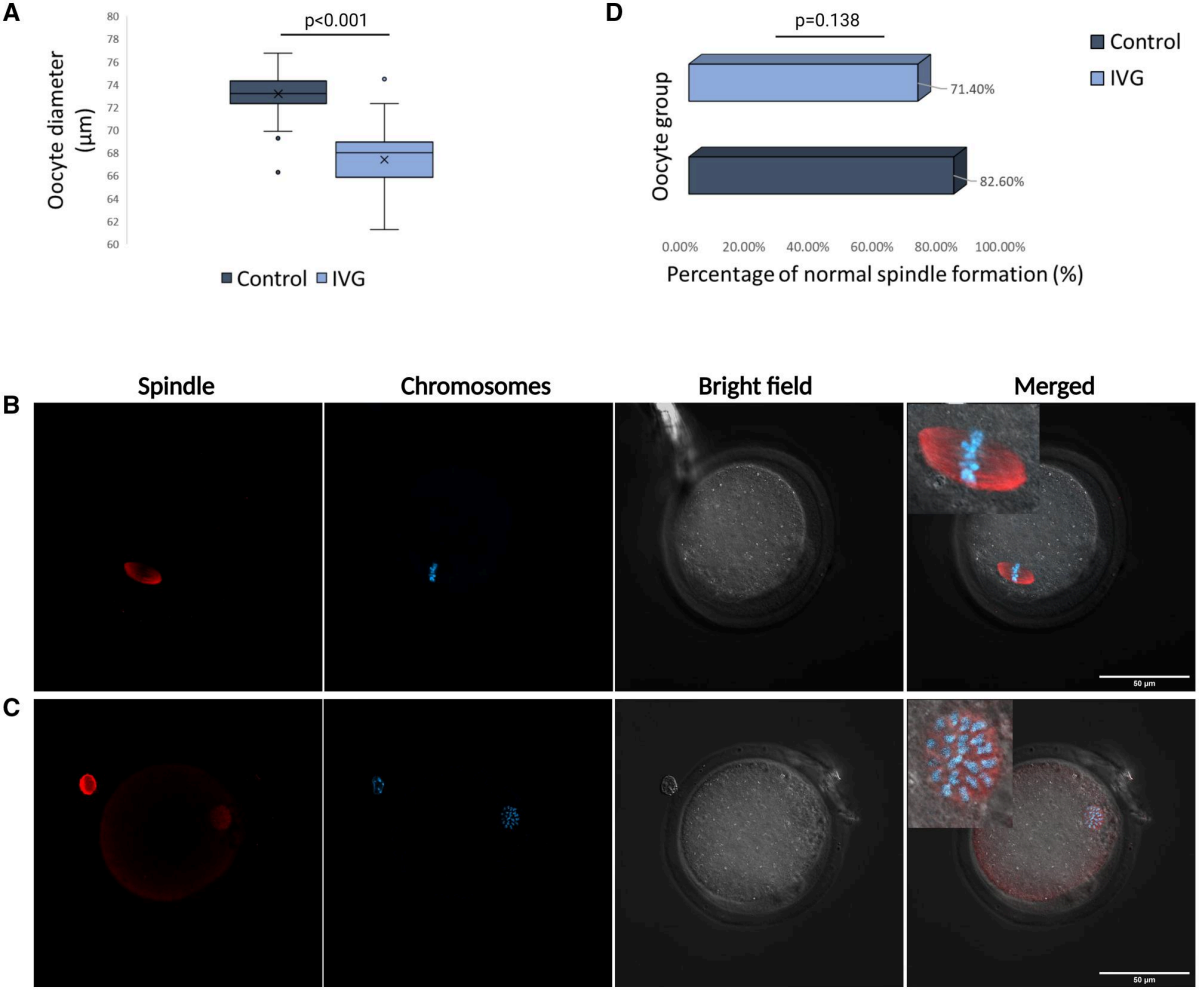
**Oocyte diameter and spindle staining in IVG and control groups**. (**A**) Average diameter of mature IVG and control oocytes from B6D2 mice, measured under an inverted bright field microscope. Difference between means was calculated with independent Student’s *t*-test. *P* < 0.05 was considered significant. (**B**) Mature mouse oocyte with normal spindle morphology, imaged with confocal microscopy. An enhanced image of the spindle structure can be found in the upper left corner of the merged image. (**C**) Mature mouse oocyte with abnormal spindle structure morphology. An enhanced image of the spindle structure can be found in the upper left corner of the merged image. The spindle is visualized in red and chromosomes in blue. Scale bars: 50 μm. (**D**) Percentage of normal spindle morphology in IVG and control oocytes. Difference between groups was calculated with chi-square (χ^2^) analysis. *P* < 0.05 was considered significant. IVG: *in vitro*-grown.

Following oocyte maturation, IVG oocytes were compared to control, *in vivo*-grown oocytes collected from stimulated B6D2 females. Diameters from 72 IVG and 85 control MII oocytes were calculated using ImageJ. The average oocyte diameter in the IVG group was 67.4 μm, which was significantly lower when compared to control oocytes, measuring 73.1 μm (*P* < 0.001, independent Student’s *t*-test, Fig. 2A), suggesting poor oocyte growth. In order to assess nuclear maturation normalcy, we performed spindle–chromosome-complex staining. Oocytes were stained with anti-a and b-tubulin (spindle structure) and Hoechst 33258 (chromosomes). A normal spindle was defined by the presence of bipolar ends, a barrel shape and chromosomes aligned in the centre of the structure (Fig. 2B). Spindles with any abnormalities in tubulin and/or chromosomes were categorized as abnormal (Fig. 2C). In the control group, 62/75 (82.6%) oocytes had a normal spindle morphology compared to 35/49 (71.4%) in IVG oocytes (Fig. 2D). The difference was not statistically different between the two groups (*P* = 0.138, χ^2^ test).

### Calcium-releasing ability and mitochondrial membrane potential are compromised in IVG oocytes

**Figure 3.**
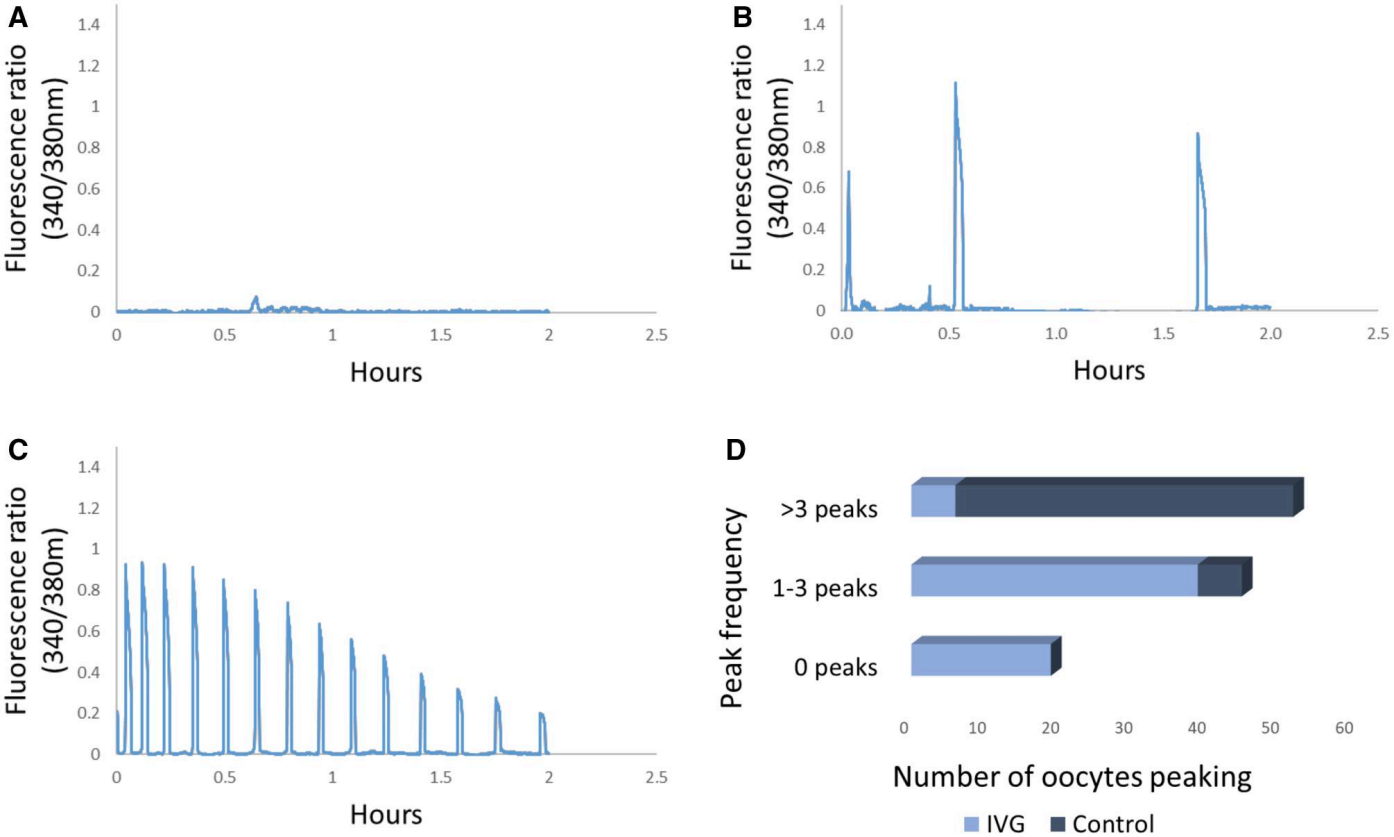
**Calcium imaging analysis of IVG and control groups**. (**A**) Oocyte showing no calcium release (0 peaks). (**B**) Oocyte showing calcium release with three distinct peaks. (**C**) Oocyte with several calcium peaks. (**D**) Frequency of calcium peaks in IVG and control oocytes. IVG: *in vitro*-grown.

Some of the mature oocytes from the IVG culture and control oocytes were analysed for calcium-releasing ability following parthenogenetic activation with SrCl_2_. Oocytes were categorized according to the peak frequency, with oocytes producing 0 (Fig. 3A), 1–3 (Fig. 3B) and >3 (Fig. 3C) peaks. In total, 19/64 IVG oocytes did not release any calcium (Fig. 3D), 39/64 produced 1–3 and 6/64 > 3 peaks (Fig. 3D). In contrast to the IVG group, all of the control oocytes (52/52) peaked, with the majority releasing >3 peaks (Fig. 3D). The average amplitude (A) and frequency (F) of calcium peaks were calculated for each group (Table 1), based on the ratio of 340/380 nm. The product of AxF represents indirectly the total calcium release. For IVG oocytes, the calcium release was 1.62, which was significantly lower (*P* < 0.001) than the control oocytes (5.74) (Table 1).

**Table 1.** Calcium-releasing ability of *in vitro*-grown and control mouse oocytes.

	IVG	Control
Analysed oocytes	64	52
Average A	0.61	0.67
Average F	2.65	8.56
A×F (AU)	1.62*	5.74

Oocyte activation was performed following exposure to SrCl_2_. The average amplitude (A) value and average frequency (F) for each group are displayed. The product of A×F represents the calcium release. Values of A×F were compared with Mann–Whitney *U* test. Differences with a *P* value <0.05 were considered significant. Asterisks represent a *P* value <0.05 between IVG and control oocytes.

* *P* < 0.001.

IVG: *in vitro*-grown; AU: arbitrary units.

**Figure 4.**
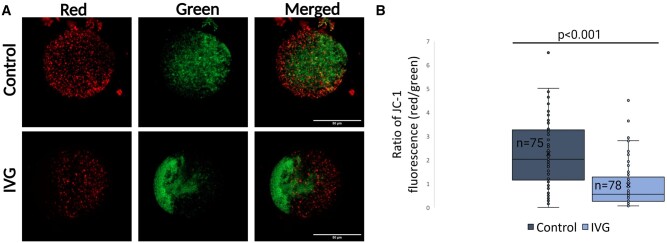
**Mitochondrial membrane potential in control and IVG metaphase II mouse oocytes**. (**A**) Representative images displaying control (upper panel) and IVG metaphase II oocytes (lower panel), after incubation with JC-1 dye. Red fluorescence represents JC-1 aggregates and green fluorescence JC-1 monomers, respectively. Scale bars: 50 μm. (**B**) Ratio of JC-1 fluorescence, representing mitochondria membrane potential, in control and IVG oocytes. *N*: number of oocytes used for each group. Difference between means was calculated with Mann–Whitney non-parametric test. *P* < 0.05 was considered significant. IVG: *in vitro*-grown.

In addition to calcium-releasing ability, mitochondrial inner membrane potential was evaluated after oocytes were exposed to the JC-1 dye. Emitted green fluorescence represents mitochondria with poor membrane potential (ΔΨm <100 mV) and red fluorescence mitochondria with higher membrane potential (ΔΨm >140 mV). Representative images are shown in Fig. 4A for control and IVG oocytes. The ratio of red/green fluorescence was calculated to indirectly estimate the mitochondrial membrane potential between the two groups (Fig. 4B). In total, the membrane potential was estimated in 78 IVG and 75 control oocytes. The average value in IVG oocytes was 0.95, severely compromised and significantly lower compared to *in vivo*-grown controls (2.27, *P* < 0.001).

### Poor embryo development in IVG oocytes could be overcome by spindle and PNT in parthenogenetically activated oocytes

In order to compare the developmental potential between the two groups, we first generated diploid parthenogenetic embryos by exposing IVG and control MII oocytes to SrCl_2_ and cytochalasin D for 4 h. Following 4 h, 37/51 (72.5%) IVG and 57/58 (98.2%) control oocytes survived and were kept in culture to evaluate embryo development. In total, 22/37 (59.4%) oocytes cleaved to two cells in the IVG group, significantly lower than in the control group (51/57(89.5%)) (*P* < 0.001, Table 2). Blastocyst development was also impaired in the IVG group, with only 8/22 (36.3%) of two-cell embryos reaching the blastocyst stage in contrast to 45/51 (88.2%) in the control group (*P* < 0.001, Table 2).

**Table 2.** Development of parthenogenetically activated mouse embryos.

Groups	No oocytes/zygotes	No of reconstructed oocytes/zygotes	Fused reconstructed oocytes/zygotes	Two-cell (%)	Blastocysts (%)
Control	57			51/57 (89.5%)	45/51 (88.2%)
IVG	37			22/37 (59.4%)**	8/22 (36.3%)**
ST-IVG	34	25/34 (73.5%)	18/25 (72%)	16/18 (88.8%)^A^	16/16 (100%)^C^
ST-control	22	22/22 (100%)	16/22 (72.7%)	16/16 (100%)^B^	14/16 (87.5%)^B^
PNT-IVG	20	20/20 (100%)	19/20 (95%)	19/19 (100%)^A^	17/19 (89.4%)^C^
PNT-control	14	14/14 (100%)	13/14 (92.8%)	11/13 (84.6%)	11/11 (100%)^B^

Oocyte activation took place following exposure to SrCl_2_. The blastocyst rate was calculated based on the number of oocytes/zygotes that cleaved (two-cell rate). Comparison was made using chi-square test, with *P* < 0.05 considered statistically significant. Within the same column, the percentages with asterisk (**) mark a statistical significance compared to control.

** *P* < 0.001.

Percentages with superscript uppercase letters mark a statistical significance compared to the IVG group.

^A^ *P* < 0.05.

^B^ *P* < 0.01.

^C^ *P* < 0.001.

IVG: *in vitro*-grown; PNT: pronuclear transfer; ST: spindle transfer.

**Figure 5.**
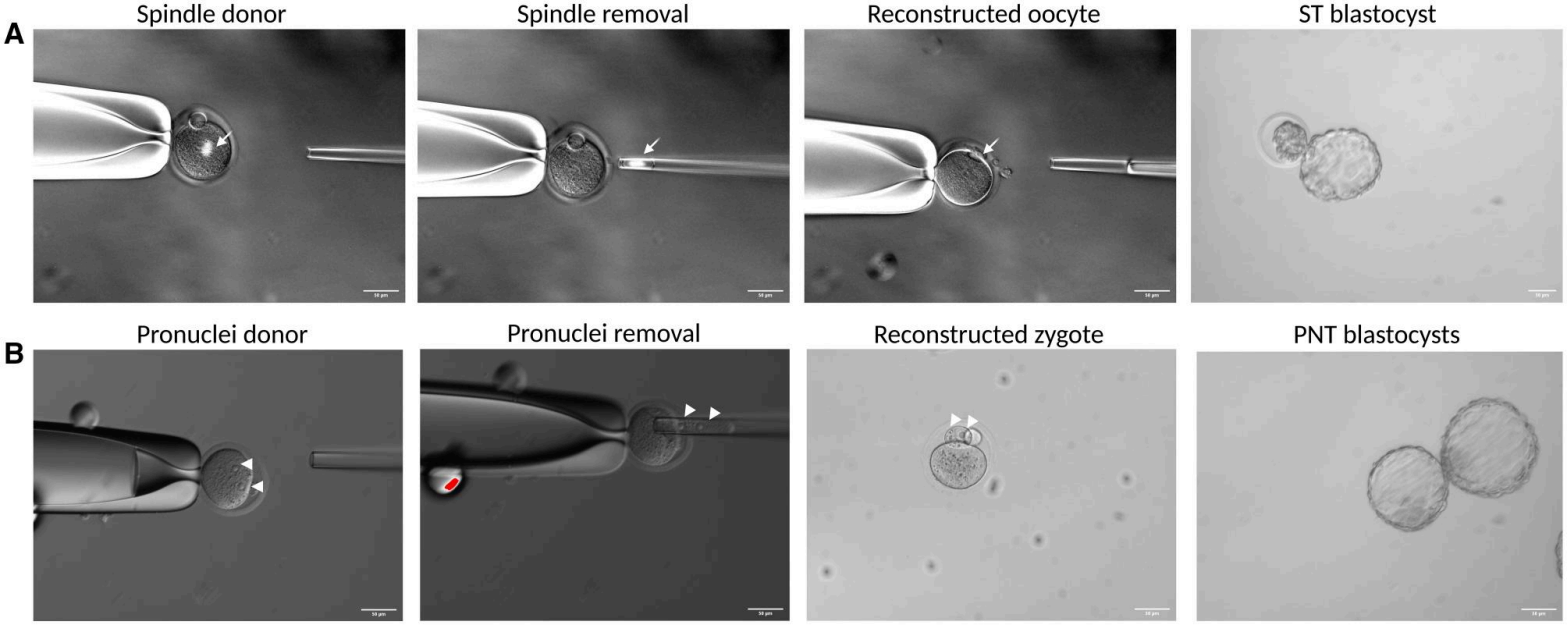
**Representation of ST and PNT procedures**. (**A**) ST representation: the spindle from a spindle donor is removed and transferred to an enucleated recipient. ST blastocyst: blastocyst originating from ST reconstructed oocytes. Spindles are indicated with white arrows. (**B**) PNT representation: the pronuclei from a pronuclei donor are removed and transferred in an enucleated recipient zygote. PNT blastocyst: blastocyst originating from PNT reconstructed zygotes. White arrowheads indicate the pronuclei. Scale bars: 50 μm. ST: spindle transfer, PNT: pronuclear transfer.

After confirming poor embryo development and compromised cytoplasmic quality in the IVG group based on the calcium and mitochondrial staining data, we decided to perform ST. Spindles from IVG or control oocytes (spindle donor, Fig. 5A) were removed and transferred to the cytoplasm of enucleated control oocytes (ST-IVG and ST-control group, respectively). Successfully reconstructed and fused oocytes (Table 2) were exposed to SrCl_2_ for 4 h (Fig. 5A). All reconstructed oocytes survived following exposure to SrCl_2_ and embryo development was monitored until the blastocyst stage (Fig. 5A). The two-cell rate significantly improved from 59.4% (22/37) in IVG oocytes to 88.8% (16/18) in ST-IVG oocytes (*P* < 0.05), which was similar to ST-control and control oocytes (Table 2). Blastocyst development was also significantly increased, reaching 100% in the ST-IVG group (*P* < 0.001, Table 2), comparable to *in vivo*-grown controls.

For PNT, IVG and control oocytes were first exposed to SrCl_2_ for 4 h. Following 5 h, PNs were visible and PNT could be performed, with a high reconstruction and fusion rate (Table 2). The two PNs from IVG parthenogenetically activated oocytes or from parthenogenetically activated control zygotes (PNs donor, Fig. 5B) were transferred (Fig. 5B) to the cytoplasm of enucleated parthenogenetically activated control oocytes (PNT-IVG group and PNT-control group, respectively). Reconstructed zygotes were monitored up to the blastocyst stage (Fig. 5B). Following PNT, a similar pattern was seen as in the ST embryos, since PNT significantly improved embryo development, generating a 89.4% (17/19) blastocyst rate, and these results were similar to the control groups (Table 2). In short, these data demonstrate that both ST and PNT were able to rescue poor embryo development of IVG oocytes after parthenogenetic activation and restore it to levels similar to controls.

### Application of PNT in biparental embryos improves embryo development

In order to decipher if healthy pups could be generated from this approach, we created biparental mouse embryos. Initially, we attempted to fertilize the IVG oocytes using Piezoelectric Intracytoplasmic Sperm Injection (PIEZO-ICSI), but the sensitivity of the IVG oocytes led almost to complete degeneration, likely due to inherent quality. As such, we continued with IVG zygotes and not IVG oocytes, and, for this purpose, we decided to proceed with PNT. We cultured 2680 additional follicles from 15-day-old B6D2 mice, while 1515 COCs from stimulated mice were included as controls. The COCs from IVG and control groups were incubated for 4 h with sperm from B6D2 males. Zygotes were thoroughly washed and were examined, following 5 h post sperm exposure, under an Olympus microscope. Zygotes were classified as 2PN when two PNs and two polar bodies were present (normal fertilization). From the 1090 isolated COCs in the IVG culture, only 15.5% showed the formation of two PNs (Table 3). On the contrary, a significantly higher 2PN rate was observed in the control group (*P* < 0.001), reaching 45.5% (Table 3). In total, 371 control and 40 IVG zygotes were left in culture to monitor embryo development. In the control group, 95.4% of zygotes reached the two-cell stage, compared to only 42.5% in the IVG group (Table 3). Moreover, blastocyst development was severely compromised in IVG zygotes, as no blastocysts were formed, compared to an 86.2% blastocyst rate in zygotes from control oocytes (*P* < 0.001, Table 3). Since poor embryo development was observed in the IVG group, we decided to use the rest of the formed zygotes for PNT.

**Table 3.** Development of biparental mouse embryos following IVF.

Groups	No of COCs	Two pronuclei (%)	Number of reconstructed zygotes	Fused reconstructed zygotes	Two-cell (%)	Blastocysts (%)
Control	1515	690/1515 (45.5%)			354/371 (95.4%)	320/371 (86.2%)
IVG	1090	170/1090 (15.5%)**			17/40 (42.5%)***	0/40 (0%)***
PNT-IVG			84/84 (100%)	84/84 (100%)	79/84 (93.5%)^C^	73/84 (86.9%)^C^
PNT-control			84/84 (100%)	84/84 (100%)	84/84 (100%)^C^**	81/84 (96.4%)^C^*

Pronuclei formation took place following fertilization for 4 h with sperm from B6D2 males. The blastocyst rate was calculated based on the number of oocytes that were normally fertilized (two pronuclei). Comparison was made using chi-square test (χ^2^), with *P* < 0.05 considered statistically significant. Within the same column, the percentages with asterisk (*) mark a statistical significance compared to control.

* *P* < 0.05.

** *P* < 0.01.

*** *P* < 0.001.

Percentages with superscript uppercase letters mark a statistical significance compared to the IVG group.

^C^ *P* < 0.001.

IVG: *in vitro*-grown; PNT: Pronuclear transfer.

PNs from IVG zygotes were transferred to the cytoplasm of enucleated *in vivo* MII oocytes fertilized using IVF (referred to as control zygotes). In addition, we included a PNT-control group, where PNs from control zygotes were transferred to enucleated control zygotes. In total, 84 PNT-IVG and 84 PNT-control zygotes were successfully reconstructed and fused (Table 3) and embryo development up to the blastocyst stage was monitored. Approximately 94% of the PNT-IVG zygotes reached the two-cell stage and 100% in the PNT-control group. The two-cell rate in PNT-IVG zygotes was similar to the two-cell rate observed in the IVF-control group but significantly higher to that of IVG oocytes (*P* < 0.001, Table 3). Blastocyst development was significantly increased in PNT-IVG (86.9%) and PNT-control (96.4%) groups when compared to IVG zygotes (*P* < 0.001 and 0.001, respectively, Table 3). Most importantly, blastocyst formation was similar between PNT-IVG and IVF-control groups (Table 3).

### Genetic analysis of generated embryos proves that PNT does not induce chromosomal errors and supports live birth of fertile offspring

Following culture up to Day 3.5, some blastocysts following IVF from control (n = 17), PNT-IVG (n = 13) and PNT-control (n = 16) embryos were assessed for chromosomal abnormalities, using shallow whole genome sequencing. Fourteen of the analysed embryos in the control group had a normal CNV profile (Supplementary Fig. S1A), while three had chromosomal duplications. In the PNT-control group, 16 embryos were analysed. In four of the embryos, the sequencing failed. From the remaining 12 embryos, 10 presented a chromosomally normal profile (Supplementary Fig. S1A). From the PNT-IVG group, a total of 13 embryos underwent genetic analysis. Three of the embryos failed in sequencing, while from the remaining 10 embryos, 8 had a normal chromosomal profile (Supplementary Fig. S1A).

The remaining created embryos were vitrified and warmed for embryo transfer. An average of 15 embryos was warmed for every embryo transfer, whilst different surrogate CD1 females were used for every group. In total, eight pups were born (four males and four females) from the control group, five from the PNT-control (four males and one female) and six from the PNT-IVG group (four males and two females) (Supplementary Fig. S1B–D). In order to assess the fertility of the derived pups, we mated some of the female and male PNT-control (n = 3) and PNT-IVG (n = 4) derived mice with control B6D2 mice (n = 6). This mating resulted in many healthy pups (n = 45) (Supplementary Table S1).

## Discussion

To our knowledge, this is the first time that PNT has been applied to overcome poor embryo development of mature IVG oocytes originating from secondary mouse follicles. Mouse follicle culture has been extensively studied by several groups, and generation of IVG oocytes from the most immature follicular stage has been possible since 1996 (Eppig *et al.*, 1996). Although culture settings have been significantly improved, leading to high rates of oocyte/embryo development and live offspring, the potential of IVG oocytes remains inferior to their *in vivo*-grown counterparts (Kim *et al.*, 2004; Xu *et al.*, 2006; Obata *et al.*, 2007; Guzel and Oktem, 2017; Takashima *et al.*, 2021), which was also confirmed by our study.

Here, we characterized IVG oocytes grown in V-bottom well plates. Our goal was not to improve current culture conditions, but to assess the quality of the IVG oocytes and investigate means to overcome poor embryo development. As a first step, we assessed follicular growth following 9 days of culture. In contrast to other 3D culture systems, evident antral follicle formation was not achieved. Nevertheless, a 3D morphology was not supported in the case of mouse follicles, which diffused following 3 days of culture, leading to cell attachment to the plate and rupture of the basal membrane, as observed in 2D cultures (Sánchez *et al.*, 2012).

The maturation rate reported here, at ∼76%, is similar to those reported in the literature (Jin *et al.*, 2010; Herta *et al.*, 2022). Despite sufficient nuclear maturation, IVG oocytes exhibited compromised embryo development compared to *in vivo*-grown oocytes from stimulated mice. Spindle formation did not seem to be the reason for this evident developmental arrest since a normal rate of spindle–chromosome structures was observed in the majority of the analysed IVG oocytes, similar to *in vivo*-grown oocytes. However, it should be stated that genetic profiles from IVG oocytes, which would be the ultimate characterization for chromosomal abnormalities, were not analysed, owing to limitations in the reliable analysis of single cells.

Taking into consideration that the developmental competence of the oocyte is associated with the follicle diameter and antral formation (Coticchio *et al.*, 2015; Simon *et al.*, 2020), we measured the diameters of the IVG oocytes, which were significantly lower compared to those observed in the *in vivo* control group. Sufficient cytoplasm should be available, since the oocyte is a store for mRNAs, mitochondria and proteins, which regulate the key events of fertilization and embryonic cleavage (Mao *et al.*, 2014; Coticchio *et al.*, 2015; Reader *et al.*, 2017). Importantly, calcium release and mitochondrial membrane potential were also compromised in the studied group. Irregularities in the calcium-releasing machinery of the oocyte might lead to improper fertilization and embryo arrest (Yeste *et al.*, 2017), since calcium is important for the exit from the meiotic arrest, proper formation of PNs, recruitment of maternal mRNAs and embryo development (Zafar *et al.*, 2021). Moreover, mitochondria are important organelles for energy production and key factors for successful embryo growth. Mitochondrial replication does not occur earlier than the blastocyst stage in embryos and, as such, the initial numbers and functionality of available mitochondria in the oocyte will cover the energy demands of fertilization and embryo development (Coticchio *et al.*, 2015). Previously, compromised oocyte growth, calcium release, mitochondrial membrane potential and mitochondrial numbers have been reported in the literature in IVG mouse oocytes, and in several culture systems (Li *et al.*, 2021; Takashima *et al.*, 2021; Paulino et al., 2022). Our results could therefore explain the poor embryo development observed while they further support the cytoplasmic inferiority of the IVG oocytes. In an attempt to improve the embryonic growth of our mouse IVG oocytes, we implemented NT.

PNT was able to improve the evident embryo developmental arrest, increasing blastocyst development to a similar rate as observed in the *in vivo*-grown control oocytes. Although ST stands as the preferred NT technique, it was not adopted further than parthenogenetically activated oocytes in this study. This decision was influenced by the observed fragility of the IVG oocytes in our culture system, displaying complete degeneration following PIEZO-ICSI in our initial pilot experiments. As such, IVG biparental embryos could not be generated. Since one of our initial goals was to compare IVG biparental embryonic development with ST-IVG biparental embryos, we could not continue further with this approach. However, ST could have also been performed to overcome poor embryo development. NT has been used in the past successfully to overcome cytoplasmic inferiority of IVG oocytes from primordial germ cells and early preantral follicles (Obata *et al.*, 2002, 2007). However, oocytes exhibited a low maturation rate, and germinal vesicle transfer was necessary in order to achieve maturation, using *in vivo*-grown GV oocytes as cytoplasmic recipients. Conversely, following maturation, a poor fertilization rate was achieved, as well as limited blastocyst formation. A second NT was necessary, including the transfer of the spindle from the reconstructed *in vitro* matured oocytes to the cytoplasm of *in vivo* matured oocytes, before fertilization and embryo development were restored similarly to controls (Obata *et al.*, 2002, 2007). Yet, the approach of serial NT, although promising, demands a higher number of available oocytes from donors, which might not be realistic for clinical application. Here, we provide evidence that PNT could also be considered as an approach to overcome cytoplasmic inferiority of IVG oocytes, supporting normal blastocyst formation rates and giving rise to live fertile offspring.

We recently confirmed the applicability of NT technology, although this was with the implementation of ST, for *in vitro* matured oocytes originating from transgender men under testosterone treatment (Christodoulaki *et al.*, 2023). It was previously reported that these oocytes exhibit low fertilization and embryo potential (Lierman *et al.*, 2021), probably owing to cytoplasmic factors, which nevertheless was partially overcome by the application of ST (Christodoulaki *et al.*, 2023). The technology of NT has also been considered for certain infertility indications, including failed fertilization and embryo developmental arrest in both animal and human studies (Zhang *et al.*, 2016; Costa-Borges *et al.*, 2020; Tang *et al.*, 2020, 2022). Until recently, the only live birth in human from NT was reported by Zhang *et al.* (2017) in a case study involving a patient with a mitochondrial DNA mutation, leading to a healthy boy. In addition, Costa-Borges *et al.* (2023) reported the birth of six more children following the application of ST for female infertility indications. Pilot studies are also currently being carried out in the UK and Ukraine, but no peer reviewed papers have been published yet (Cohen *et al.*, 2020).

In our study, we observed that PNT does not increase chromosomal aberrations in the derived embryos as well as that PNT-derived pups are fertile. This observation should be considered with caution as the number of embryos analysed was limited. Still, normal ploidy rates and live births have been reported in other studies (Obata *et al.*, 2002; Tang *et al.*, 2019, 2020; Costa-Borges *et al.*, 2020).

Long culture protocols have been established in order to achieve the antral stage and oocyte maturation, which are known to have an adverse effect not only on the quality of oocyte and embryo development in animal species (Xu *et al.*, 2006; Obata *et al.*, 2007) but also on their metabolic, transcriptomic and epigenetic profiles (Saenz-de-Juano et al., 2020; Takashima *et al.*, 2021; Herta *et al.*, 2022). In the current study, we confirmed that the poor oocyte quality observed in our culture system was attributed to cytoplasmic inferiority of the IVG oocytes and that PNT is a suitable candidate to overcome poor embryo development for this indication. Furthermore, the observation that nuclear maturation was possible despite the absence of complete antral formation could be a promising approach for human studies, where follicles of variable diameters can be collected during ovarian tissue cryopreservation and full-term antral support remains a challenge. PNT could be applied as a final step for IVG human follicles, overcoming the poor oocyte quality caused by *in vitro* extended culture, although confirmation with human studies is currently missing. Additionally, exploring the application of ST in human IVG oocytes holds potential as a strategy to obviate the necessity for utilizing zygotes, a requirement in the context of PNT, and as means to potentially increase fertilization rate of the reconstructed oocytes, which was poor in IVG oocytes.

## Supplementary Material

hoae009_Supplementary_Figure_S1

hoae009_Supplementary_Table_S1

## Data Availability

The data underlying this article are available in the article and in its online Supplementary Material.
