# Supplementary material for: Pronuclear transfer rescues poor embryo development of in vitro-grown secondary mouse follicles
Source: Hum Reprod Open. 2024 Feb 10;2024(1):hoae009. doi: 10.1093/hropen/hoae009 (PMC10904147; doi:10.1093/hropen/hoae009)
Supplement: hoae009_Supplementary_Figure_S1 [file hoae009_supplementary_figure_s1.docx]

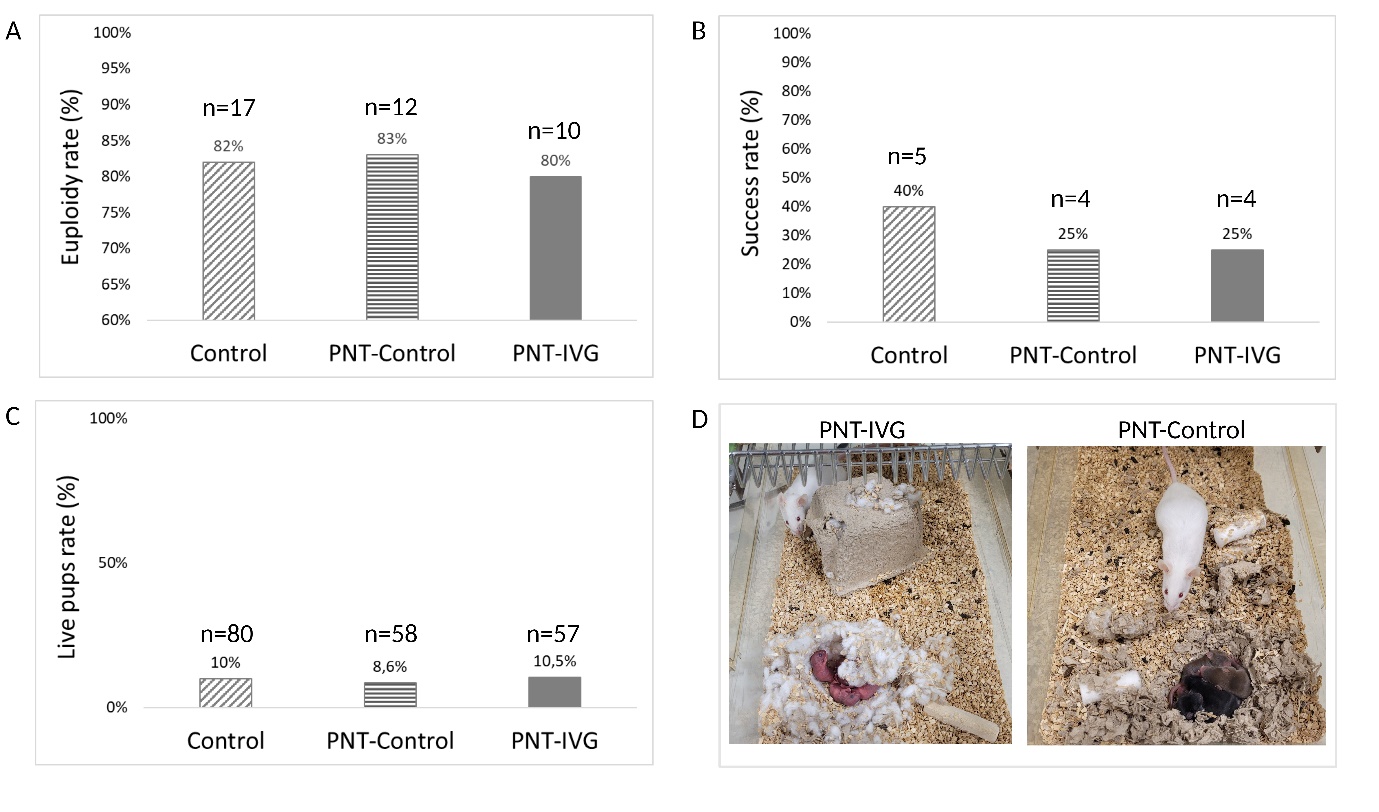
**Supplementary Figure S1** Euploidy rate of derived embryos and success rate of living pups in Control, PNT-Control and PNT-IVG groups.

**A)** Euploidy rate of analysed blastocysts from Control, PNT-Control and PNT-IVG groups. The number of analysed blastocysts is mentioned on top of each column. **B)** Success rate of embryo transfers, calculated from the number of embryo transfers that led to living offspring, to the total number of transfers performed/group. The total number of embryo transfers/group is mentioned on top of each column. **C)** Live pups rate calculated from the total number of pups to the total number of embryos transferred. The total number of embryos transferred is mentioned for every group. **D)** One-day-old PNT-IVG pups and one-week-old PNT-Control pups with their surrogate CD1 mothers.

PNT: Pronuclear transfer, IVG: *In vitro* grown
