# Supplementary material for: Pronuclear transfer rescues poor embryo development of in vitro-grown secondary mouse follicles
Source: Hum Reprod Open. 2024 Feb 10;2024(1):hoae009. doi: 10.1093/hropen/hoae009 (PMC10904147; doi:10.1093/hropen/hoae009)
Supplement: hoae009_Supplementary_Table_S1 [file hoae009_supplementary_table_s1.docx]

**Supplementary Table S1**: **Pups born following mating of Pronuclear transfer derived mice**

| **Group** | **PNT-Control females** | **PNT-Control males** | **PNT-IVG**  **females** | **PNT-IVG**  **males** |
| --- | --- | --- | --- | --- |
| **Number of mice mated/group** | 1 | 2 | 2 | 2 |
| **Number of pups born** | 9 | 15 | 3 | 18 |
| **Number of females** | 7 | 8 | 3 | 12 |
| **Number of males** | 2 | 7 | 0 | 6 |

IVG: *in vitro* grown, PNT: Pronuclear transfer
